# Failure to follow medication changes made at hospital discharge is associated with adverse events in 30 days

**DOI:** 10.1111/1475-6773.13292

**Published:** 2020-05-20

**Authors:** Daniala L. Weir, Aude Motulsky, Michal Abrahamowicz, Todd C. Lee, Steven Morgan, David L. Buckeridge, Robyn Tamblyn

**Affiliations:** ^1^ Department of Epidemiology and Biostatistics, Department of Medicine, McGill University Montreal Quebec Canada; ^2^ Clinical and Health Informatics Research Group Department of Medicine McGill University Montreal Quebec Canada; ^3^ Research Center Centre hospitalier de l’Université de Montréal Montreal Quebec Canada; ^4^ Department of Management, Evaluation & Health Policy School of Public Health Université de Montréal Montreal Quebec Canada; ^5^ Research Institute of the McGill University Health Centre Montreal Canada; ^6^ School of Population and Public Health Faculty of Medicine University of British Columbia Vancouver British Columbia Canada

**Keywords:** adherence, adverse health outcomes, hospitalization, medication changes

## Abstract

**Objective:**

To evaluate the hypothesis that nonadherence to medication changes made at hospital discharge is associated with an increased risk of adverse events in the 30 days postdischarge.

**Study Setting:**

Patients admitted to hospitals in Montreal, Quebec, between 2014 and 2016.

**Study Design:**

Prospective cohort study.

**Data Collection:**

Nonadherence to medication changes was measured by comparing medications dispensed in the community with those prescribed at hospital discharge. Patient, health system, and drug regimen‐level covariates were measured using medical services and pharmacy claims data as well as data abstracted from the patient's hospital chart. Multivariable Cox models were used to determine the association between nonadherence to medication changes and the risk of adverse events.

**Principal Findings:**

Among 2655 patients who met our inclusion criteria, mean age was 69.5 years (SD 14.7) and 1581 (60%) were males. Almost half of patients (n = 1161, 44%) were nonadherent to at least one medication change, and 860 (32%) were readmitted to hospital, visited the emergency department, or died in the 30 days postdischarge. Patients who were not adherent to any of their medication changes had a 35% higher risk of adverse events compared to those who were adherent to all medication changes (1.41 vs 1.27 events/100 person‐days, adjusted hazard ratio: 1.35, 95% CI: 1.06‐1.71).

**Conclusions:**

Almost half of all patients were not adherent to some or all changes made to their medications at hospital discharge. Nonadherence to all changes was associated with an increased risk of adverse events. Interventions addressing barriers to adherence should be considered moving forward.

**What This Study Adds**

- A number of medication changes occur during hospitalization.
- What is not known is whether non‐adherence to these medication changes will increase the risk of adverse events post‐discharge.
- We found that almost half of patients were nonadherent to at least one medication change made at discharge in the 30 days posthospitalization.
- We also found that patients who were not adherent to any of their medication changes had a significantly higher risk of adverse events compared to those who were adherent to all changes.

## 1 INTRODUCTION

Historically, hospitals have been organized to respond rapidly and efficiently to acute illness or injury. However, institutions are now increasingly managing high‐risk, older patients who frequently require repeated admissions to hospital for exacerbations of their chronic conditions.^1^ Approximately one third of multimorbid patients discharged from an acute care hospital are readmitted within 90 days, and each additional chronic condition independently increases the risk of such short‐term readmissions.^2^, ^3^, ^4^, ^5^

Identifying strategies to prevent readmissions in complex patients is challenging because the reasons for returning to hospital can include many interlinked patient, health provider, and system level factors.^6^ The impact of patient medications is of significant interest in this area since a large proportion of readmissions are related to adverse drug events (ADEs).^7^ Severe ADEs contribute to 20% of all hospitalizations in the elderly with an associated cost of over 900 million dollars in the United States^8^ and 36 million dollars per year in Canada.^9^ One study of hospitalized patients found that 11% of elderly patients experienced an ADE postdischarge, of which 27% were considered preventable and 33% ameliorable.^10^

A number of studies have demonstrated that when older adults are hospitalized, they are often discharged on substantially different medication regimens than those at admission.^11^, ^12^, ^13^ One might expect that discontinuations, additions, or modifications to patient drug regimens during hospitalization will reduce the likelihood of readmission in so far as patients actually follow these changes. However, a few studies have found that a substantial proportion of patients do not adhere to the intended medication regimen prescribed at discharge.^14^, ^15^, ^16^, ^17^ What is not known is whether nonadherence to these medication changes will increase the risk of adverse events postdischarge, and the extent to which any change in risk may be modified by the type of medication change.

We had a unique opportunity to evaluate the impact of nonadherence to medication changes made at hospital discharge on the occurrence of adverse events by linking comprehensive information on medications dispensed in the community and those prescribed at hospital discharge with adverse events in a universal health system.

## 2 METHODS

### 2.1 Study design & setting

This prospective cohort study was a secondary analysis of a cluster randomized trial of medication reconciliation (The RightRx Trial) conducted in the province of Quebec, where comprehensive data are available on all medical visits, emergency department (ED) visits, hospitalizations, long‐term care admissions, deaths, and medications dispensed in the community. We linked individual data from the randomized trial with comprehensive provincial data for consenting patients.^18^, ^19^ The RightRx study was conducted at the McGill University Health Centre (MUHC)—a consortium of five tertiary hospitals for adults and children in Montreal, Quebec, with over 1000 beds and 36 730 admissions per year. The MUHC has a clinical information system which provides an integrated display of patient‐specific hospital information including drugs, lab results, imaging, and prior admissions. The cluster randomized trial was conducted between October 2014 and November 2016 to evaluate the effectiveness of electronic medication reconciliation in reducing medication discrepancies at discharge and adverse drug events, emergency department visits, and readmissions in the first 30 days postdischarge. All patients who were covered by provincial drug insurance and were discharged to the community or a long‐term care facility from the two internal medicine units, or the cardiac or thoracic surgery units, were eligible for inclusion in the study. The eligible study population represents 56% of all hospitalized adult patients. Patients who died during their hospital stay were not included in the analysis of study outcomes. The four units were stratified by type (medicine, surgery) and hospital location (Montreal General, Royal Victoria). The study was approved by the MUHC ethics committee (IRB #10‐180 GEN) as well as the Quebec Privacy Commissioner, and the trial was registered on clinical trials.gov (Registration # NCT01179867).^19^

### 2.2 Participants

Patients admitted from the community to medical or surgical units at the study hospitals who were older than 18 and covered under the provincial public drug plan for the year prior to admission and after discharge were eligible to be included in the current study. The province of Quebec provides health insurance (including coverage for all hospital and physician services) for all provincial residents and drug insurance to approximately 50% of all residents, including those 65 years of age and over, social assistance recipients, and those without private drug insurance (which is required by provincial regulation if an individual is not eligible for private drug insurance through their employer). If an individual has public provincial drug coverage, they are assigned to one of three drug plans based on their income. In Quebec, those with public drug coverage pay a monthly deductible of $20, as well as 35% of the costs of each medication once the deductible has been paid. The maximum amount that you can pay per month for covered medications is $91(full copay) or $54 for persons age 65 or over who receive 1% to 93% of the Guaranteed Income Supplement (partial copay). Persons who receive 94% to 100% of the Guaranteed Income Supplement do not pay any deductibles or copays (free medications). At discharge, patients who consented to be part of the trial also had to have at least one change made to their community medications at hospital discharge (excluding the prescription of new PRN medications which may not need to be filled) and be discharged home to the community or a long‐term care facility from one of the study units to be included in the current study. Patients were then followed until death or the end of the study period.

### 2.3 Data sources

For each patient, we obtained demographic, health care service use, and prescription claims data from the Quebec provincial health care administrative database (acquired for the year before hospital admission and the 1 year postdischarge). Beneficiary medical billing and pharmacy claims data have been widely validated and are frequently used for health services and epidemiologic research.^20^, ^21^, ^22^, ^23^ Community medications at admission were measured using the pharmacy claims database based on dispensations 3 months prior to admission. Medications which were likely not active at admission (ie, short courses of antibiotics) were excluded from the community medication list.

Information pertaining to the patient's hospital stay (including the discharge prescription) was abstracted from the medical chart by a trained research assistant with a clinical background. Health problems were coded using the International Classification of disease 10th revision (ICD‐10 codes), and medications were classified according to drug molecule and Anatomic Therapeutic Classification system (ATC codes).

### 2.4 Study measures

#### 2.4.1 Included medications

Medications covered through the public drug plan were included in the overall analysis, which represent most of the drugs approved for market in Canada. Medications that do not act systemically (eg, topical ointments) were excluded since they have limited capacity to affect underlying health conditions or symptoms. We also excluded over the counter (OTC) medications and those not listed on the provincial formulary since they are generally not captured in the prescription claims data. These exclusions represented 12% of all drugs prescribed at discharge (69% of all exclusions were nonsystemic medications, 29% did not have provincial drug coverage, and the remaining 2% were OTC medications) (Appendix S1: Table S1).

#### 2.4.2 Medication changes made at hospital discharge

Typically, the preadmission drug list is documented at the time of admission by the pharmacist, where they will call the community‐based pharmacy and have them fax a list of medications for a patient. This list is then validated with the patient, where changes are made as required. However, a recent study by our research team found that pharmacy claims data in the three months prior to hospital admission identified 42% more medications than were identified in the preadmission drug list found in the patients hospital chart.^23^ Therefore, for this study, community medications from prior to admission were determined using the pharmacy claims database based on dispensations 3 months prior to admission. Medications which were likely not active at admission (ie, antibiotics) were excluded from the community medication list.

At discharge, the attending physician or resident uses the current list of hospital medications as well as the community medication list (if available) to prescribe discharge medications. The patient is provided with a written discharge prescription to fill at a community pharmacy and may or may *not* receive verbal or written instructions about new medications or community medications that are being stopped or changed by a physician or nurse. If the community pharmacist has questions about whether they should continue pre‐existing medications that are not included in the discharge prescription, they ask the patient and may call the physician or discharging unit of the hospital. For our analysis, discontinued medications were defined as community medications indicated as stopped at discharge (ie, explicitly indicated as stopped in the discharge prescription). New medications were drugs that were *not* in the community medication list but were prescribed at discharge. Dose modifications were identified if a drug was present both in the community medication list and at discharge, but the corresponding prescribed daily dose differed by at least 25%. The daily dose of preadmission community medications was calculated using standard methods^20^, ^24^, ^25^, ^26^, ^27^ based on the strength, quantity, and duration fields from the pharmacy claims data. The daily dose of medications prescribed at discharge was calculated using dose per administration and frequency information abstracted from the patient chart (See Appendix S1 for additional details on dose calculations).

#### 2.4.3 Nonadherence to medication changes made at discharge from hospital

Nonadherence was assessed by comparing changes made to community medications at discharge with medications filled in the community 30 days postdischarge. Nonadherence was defined as: (a) community medications that were stopped at discharge and filled postdischarge, (b) community medications whose dose was modified at discharge but were not filled according to the modified daily dose, and (c) new medications prescribed at discharge that were not filled. For each medication, we used the drug molecule, date of hospital discharge, and beneficiary health insurance number to inspect all prescription claims records of dispensed medications in the 30 days after hospital discharge. For dose modifications, nonadherence was only considered for prescriptions that were actually dispensed. We measured nonadherence as a time‐varying variable on each day of follow‐up after discharge. The proportion of medication changes that were not adhered to (total number of medication changes not adhered to/total number of changes) was calculated for each patient on each day. Based on the value of the resulting proportion, patients were classified using a daily time‐varying indicator as: (a) adherent to all medication changes, (b) adherent to some, or (c) adherent to none of the medication changes made at discharge.

#### 2.4.4 Covariates

To control for other potential risk factors for adverse events postdischarge which may also be associated with nonadherence, we measured a number of patients, health system, and drug treatment regimen characteristics.

Patient variables measured from the Quebec health insurance demographic file included age, sex, and drug insurance type. The level of copayment required in the public drug insurance plan was included because it is associated with nonadherence and is also an indicator of socioeconomic status.^28^, ^29^

Health care utilization one year prior to the index hospitalization is associated with the risk of adverse events, including the number of preadmission hospitalizations, ED visits, ambulatory care visits, and distinct prescribers.^30^, ^31^, ^32^ Utilization characteristics were measured using preadmission medical services and prescription claims data for each patient.

Characteristics of the hospitalization that may increase the risk of adverse events include the reason for hospitalization, the unit the patient was discharged from (cardiac surgery, thoracic surgery or internal medicine), and the discharge destination (home to the community or to a long‐term care facility).^14^, ^33^ Reason for hospitalization was defined based on whether the patient was admitted for an ambulatory care–sensitive condition (admission diagnosis of grand mal status/ epileptic convulsions, chronic obstructive pulmonary disease, asthma, diabetes, heart failure and pulmonary edema, hypertension, and angina) because admissions for these conditions are a marker of poor access to appropriate primary care.^34^ For descriptive purposes, the reason for admission based on the recorded ICD‐10 code was also abstracted. Additionally, an indicator of whether patients received a discharge prescription using an electronically enabled medication reconciliation process (intervention in original trial) or usual care (control) was included.

Medication regimen characteristics that may be associated with both nonadherence and adverse events include the number of admission and discharge medications as well as the total number of medication changes made at discharge.^12^, ^30^, ^31^, ^35^ Admission medications were measured based on the number of different medications dispensed in the three months prior to hospitalization while number of discharge medications was measured based on the number of different medications in the patient's discharge prescription (from the hospital chart). Combination medications (ie, two distinct drug molecules in a single formulation) were counted as two separate ingredients. The total number of changes to community medication overall and by type (news, dose changes, stops) was also calculated.

The number and types of chronic conditions a patient had at the time of discharge were measured using the chronic conditions found in the Charlson and Elixhauser comorbidity indices^36^ using diagnostic codes in the hospital chart and medical services claims.

To understand the impact of nonadherence independent of differences in level of risk associated with patient medications, we determined the number of medications filled in the postdischarge period with a high risk of adverse effects. Drug classes with the highest risk of adverse outcomes included opiates, antibiotics, benzodiazepines, diuretics, antiepileptics, corticosteroids, anticoagulants, antidepressants, and antihypertensives.^37^ The number of high‐risk medications was measured daily as a continuous time‐varying variable based on records of dispensed prescriptions using pharmacy claims.

#### 2.4.5 Adverse health outcomes in 30 days postdischarge

The primary outcome was defined as the time to the first emergency department visit, re‐hospitalization, or death in the 30 days after discharge. Emergency department visits and hospital readmissions were determined using medical service claims that require physicians to record the date and location where the service was delivered to receive payment. Date of death was measured using the health insurance beneficiary demographic file. Reason for readmission or ED visit was based on ICD‐10 code recorded in medical services for that episode.

### 2.5 Statistical analysis

Patient characteristics were described overall and stratified by adherence. The crude incidence rate and 95% confidence intervals for the combined outcome of ED visit, readmission, or death were calculated using person‐days of follow‐up prior to the event or to the end of the 30‐day follow‐up period as the denominator. The numerator was the number of patients with at least one of these adverse outcomes in the follow‐up period. Incidence rates were calculated overall and stratified by levels of adherence.

Multivariable time‐varying Cox proportional hazards models were used to assess the potential association between levels of nonadherence to medication changes and adverse health outcomes postdischarge, while adjusting for confounders. Two separate models were fit in the primary analysis; in the first, nonadherence was modeled as a time‐varying continuous percentage, and in the second, it was categorized and represented by two time‐varying binary indicator variables: (a) adherent to none of the changes and (b) adherent to some changes, with adherent to all changes as the reference category. Model goodness‐of‐fit statistics (AIC)^38^ and change in the hazard ratio for nonadherence^39^ were used to determine which confounders to adjust for in our models.^40^ To test both the proportional hazards assumption and linearity of effects for continuous variables, we used flexible spline‐based extensions of the Cox model.^41^ Clinically relevant first‐order interaction terms with nonadherence, selected a‐priori, were formally tested.

In secondary analyses, we used similar methods to estimate three separate multivariable time‐varying Cox models, each assessing the impact of different types of nonadherence: (a) not filling new medications, (b) filling dose changes at the incorrect dose, and (c) filling discontinued medications.

### 2.6 Sensitivity analyses

A number of sensitivity analyses were conducted to assess the robustness of our analytic approach. First, we increased the follow‐up time to 90 days postdischarge to account for adverse events which may take longer to occur. We also excluded early events and started follow‐up on day 3 after discharge in order to account for potential confounding by severity of the patient's condition following discharge. Indeed, it is possible that some patients (a) may be too ill to fill their medications immediately after discharge and (b) are readmitted or visit the ED within the next 2‐3 days *because* of the severity of their condition, which could create a spurious “association” between nonadherence and very early adverse outcomes. In other words, for such patients, nonadherence is a *marker* of high risk of an adverse event, rather than its *cause*. Next, in order to confirm that the impact of nonadherence on health outcomes was the same between both arms of the original trial, we re‐conducted the primary analysis within intervention and control patients separately. Given that the total out‐of‐pocket costs patients are required to pay for their medications (rather than drug plan alone) are likely associated with both adherence and adverse health outcomes following discharge, we included total out‐of‐pocket medication costs as a confounder in our model to determine whether this impacted the association between adherence and 30‐day outcomes.

After discharge from hospital, many patients will visit their community‐based health care provider, where additional medication changes can occur. These changes could occur either as the result of adverse drug events or because the community‐based physician believes changes made to patient medications at discharge from hospital were not appropriate. In both cases, the patient will be identified as nonadherent and, at the same time, may be at increased risk of an adverse event which will *not* necessarily be caused by the nonadherence. Therefore, in an additional sensitivity analysis, the model adjusted for a time‐varying binary variable indicating that the patient had a physician visit in the community after discharge. We also adjusted for the time‐varying cumulative number of visits in a separate model. Last, we analyzed differences in the proportion of medication changes not adhered to in the time prior to and following a physician visit (among those who ultimately had a physician visit post discharge) separately to evaluate potential differences.

## 3 RESULTS

**Figure 1:**
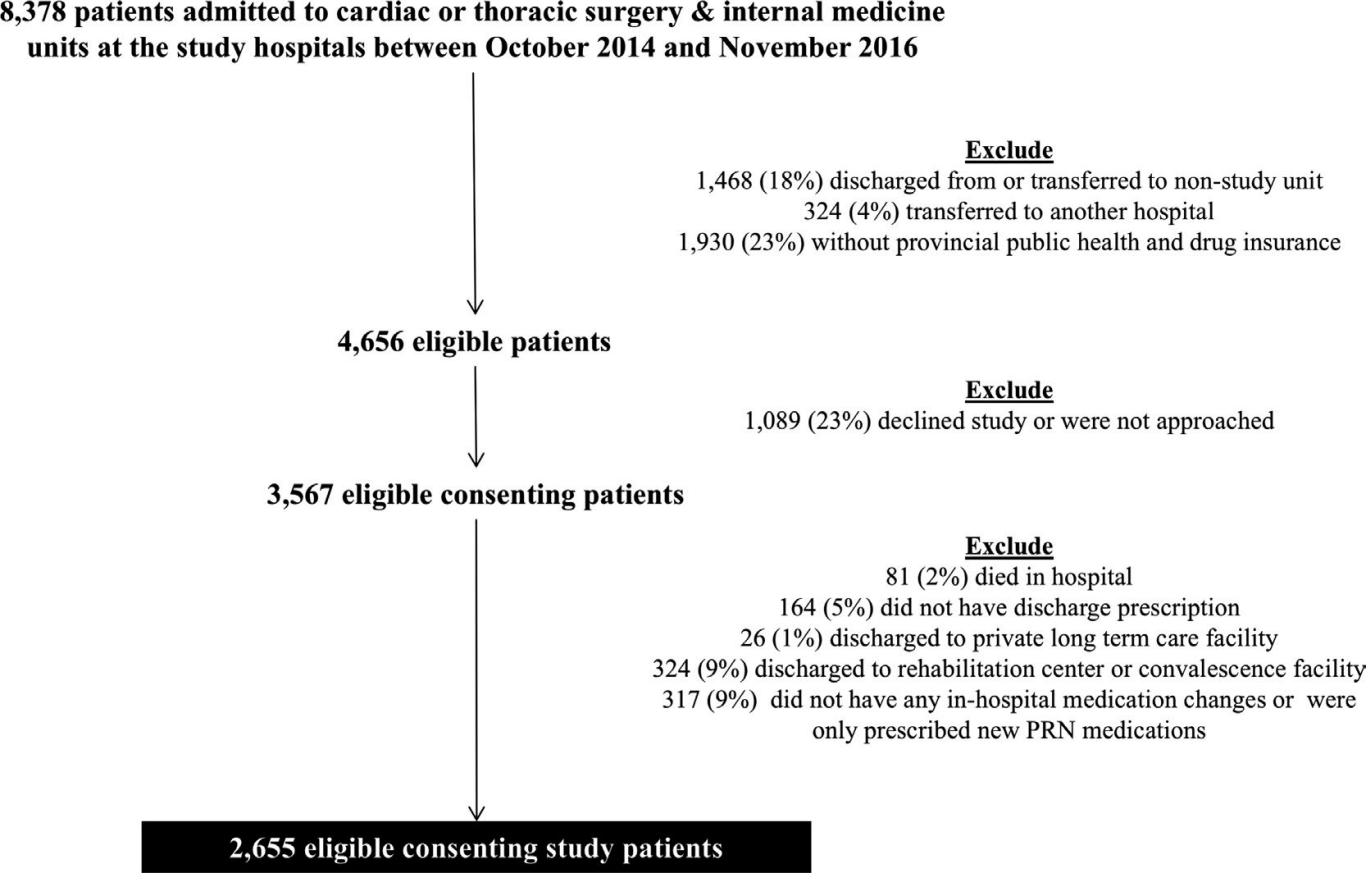
Flowchart of patient exclusions

Overall, 8378 patients were admitted to study units during the enrollment period; 1468 (14%) were not discharged from a study unit, 324 (4%) were transferred to another hospital, and 1930 (23%) did not have public provincial drug insurance; thus, 4,656 patients were eligible to be included in the original cluster randomized controlled trial. Of eligible patients, 1089 (23%) did not consent to be part of the study. An additional 81 (2%) patients died in hospital, 164 (5%) did not have a discharge prescription, 26 (1%) were discharged to a private long‐term facility, 324 (9%) were discharged to a rehabilitation or convalescence facility, and 317 (9%) did not have a change made to their medications at discharge. Thus, 2655 (74%) of consenting study patients were included in our analyses (Figure 1).

Mean age of study patients was 69.5, and 1581 (60%) were males. On average, patients had 4.6 chronic conditions, 2.8 ED visits in the 12 months prior to admission and 49% had at least one hospitalization (Table 1). The most common reasons for admission were pneumonia, heart failure, and urinary tract infections for patients from internal medicine; aortic valve stenosis, valve insufficiency, and atherosclerotic heart disease for cardiac surgery patients; and malignant neoplasms, abnormal findings on lung imaging, and other lung disorders among thoracic surgery patients. Patients were using an average of 5.8 medications at admission and were prescribed 7.9 medications at discharge, with a mean of 4.4 changes (new prescriptions, discontinuations and dose modifications) made to community medications at hospital discharge.

**Table 1:** The characteristics of 2655 eligible patients in the study population

Characteristic	
Patient demographics	N (%)
Age, mean (SD)	69.5 (14.7)
Sex	
Female	1074 (40.5)
Male	1581 (59.6)
Copay status	
Full	1562 (58.8)
Partial	594 (22.4)
Free	499 (19.8)
Health care utilization one year prior to index admission	Mean (SD)
Hospitalizations	0.9 (1.6)
Emergency department visits	2.8 (4.0)
Ambulatory care visits	12.2 (18.5)
Distinct prescribers	4.7 (3.8)
Characteristics measured during hospitalization	N (%)
Admitted for ambulatory care–sensitive condition	247 (9.3)
Number of chronic conditions, mean (SD)	4.6 (2.4)
Unit discharged from	
Internal medicine	1493 (56.2)
Cardiac surgery	687 (25.9)
Thoracic surgery	475 (17.9)
Discharge destination	
Home to community	2557 (96.3)
Long‐term care	90 (3.7)
Medication regimen characteristics	Mean (SD)
Medications at admission	5.8 (4.2)
Medications prescribed at discharge	7.9 (4.0)
Total number of medication changes	4.4 (2.6)
New medications	3.0 (2.1)
Discontinued medications	0.9 (1.3)
Dose modifications	0.6 (0.9)
Continued medications	4.3 (3.6)

The average proportion of medication changes that were not adhered to per patient over the *entire* follow‐up period was 20%; 164 (6.2%) patients were not adherent to any medication changes, 997 (37.6%) were adherent to some of the changes, and 1,494 (56.3%) were adherent to all changes during the entire follow‐up period. Patients who were not adherent to any of their medication changes were younger, had fewer chronic conditions, and were dispensed fewer medications in the 3 months prior to admission, and a larger proportion were discharged from internal medicine units compared to those who were adherent to some or all of their medication changes (Table 2). When we evaluated number of medication changes according to adherence, we found on average, those who were fully nonadherent had 3 changes (SD 2), those who were partially adherent had five changes (SD3), and those who were fully adherent had four changes (SD 2).

**Table 2:** Characteristics of 2655 patients according to nonadherence to medication changes made at hospital discharge

Patient characteristics	Proportion of medication changes not adhered to	Not adherent to any changes (n = 164)	Adherent to some changes (n = 997)	Adherent to all changes (n = 1497)
Patient demographics	Mean (SD)		N (%)	
Age				
18‐35	0.27 (0.37)	14 (8.5)	30 (3.0)	59 (4.0)
35‐64	0.22 (0.32)	50 (30.5)	230 (23.1)	377 (25.2)
65‐79	0.19 (0.28)	54 (32.9)	476 (47.7)	721 (48.3)
80+	0.21 (0.30)	46 (28.1)	261 (26.2)	337 (22.6)
Sex				
Female	0.22 (0.31)	73 (44.5)	429 (43.0)	572 (38.3)
Male	0.19 (0.30)	91 (55.5)	568 (57.0)	922 (61.7)
Copay status				
Full	0.20 (0.30)	92 (56.1)	588 (59.0)	882 (59.0)
Partial	0.19 (0.30)	29 (17.7)	232 (23.3)	333 (22.3)
Free	0.23 (0.33)	43 (26.2)	177 (17.8)	279 (18.7)
Health care utilization one year prior to index admission				
Hospitalizations				
0	0.20 (0.30)	84 (51.2)	497 (49.9)	773 (51.7)
1+	0.21 (0.30)	80 (48.8)	500 (50.2)	721 (48.3)
Emergency department visits				
0	0.19 (0.30)	45 (27.4)	247 (24.8)	459 (30.7)
1+	0.21 (0.30)	119 (72.6)	750 (75.2)	1035 (69.3)
Ambulatory care visits				
0‐3	0.18 (0.30)	45 (27.4)	191 (19.2)	409 (27.4)
4‐7	0.21 (0.30)	36 (22.0)	242 (24.3)	336 (22.5)
8‐14	0.20 (0.30)	32 (19.5)	271 (27.4)	391 (26.2)
15+	0.23 (0.31)	51 (31.1)	291 (29.2)	358 (23.1)
Distinct prescribers				
0	0.48 (0.41)	52 (31.7)	151 (15.2)	241 (16.1)
1‐2	0.15 (0.27)	31 (18.9)	253 (23.4)	451 (30.2)
3‐4	0.16 (0.25)	31 (18.9)	223 (22.4)	354 (23.7)
5+	0.21 (0.28)	50 (30.5)	370 (37.1)	448 (30.0)
Characteristics measured during hospitalization				
Reason for admission				
Non‐ambulatory care–sensitive condition	0.20 (0.30)	149 (90.9)	886 (88.9)	1373 (91.9)
Ambulatory care–sensitive condition	0.22 (0.29)	15 (9.2)	111 (11.1)	121 (8.1)
Number of chronic conditions				
0	0.26 (0.39)	11 (6.7)	14 (1.4)	45 (3.0)
1‐3	0.20 (0.32)	63 (38.4)	255 (25.6)	535 (35.8)
4‐6	0.19 (0.30)	66 (40.2)	438 (43.9)	682 (45.7)
7+	0.23 (0.28)	24 (14.6)	290 (29.1)	232 (15.5)
Types of chronic conditions				
Arrhythmia	0.19 (0.28)	33 (20.1)	321 (32.2)	420 (28.1)
Heart failure	0.23 (0.29)	33 (20.1)	299 (30.0)	287 (19.2)
Type 2 diabetes	0.23 (0.30)	57 (34.8)	431 (43.2)	460 (30.8)
Cancer, metastatic	0.19 (0.29)	21 (12.8)	120 (12.0)	211 (14.1)
Cancer, nonmetastatic	0.24 (0.32)	5 (3.1)	28 (2.8)	37 (2.5)
Chronic kidney disease	0.25 (0.31)	53 (32.3)	352 (35.3)	339 (22.7)
Liver disease	0.24 (0.34)	16 (9.8)	67 (6.7)	96 (6.4)
Lymphoma	0.21 (0.29)	4 (2.4)	51 (5.1)	58 (3.9)
COPD	0.23 (0.30)	38 (23.2)	315 (31.6)	356 (23.8)
Unit discharged from				
Internal medicine	0.26 (0.33)	135 (82.3)	629 (63.1)	729 (48.8)
Cardiac surgery	0.10 (0.20)	5 (3.1)	210 (21.1)	472 (32.6)
Thoracic surgery	0.19 (0.30)	25 (14.6)	158 (15.9)	293 (19.6)
Discharge destination				
Home community	0.20 (0.30)	159 (97.0)	931 (93.5)	1.466 (98.1)
Long‐term care	0.32 (0.30)	5 (3.1)	65 (6.5)	28 (1.9)
Drug regimen‐level characteristics				
Medications at admission				
0‐1	0.32 (0.39)	58 (35.4)	157 (15.8)	241 (16.1)
2‐4	0.15 (0.28)	36 (22.0)	174 (17.5)	448 (30.0)
5‐7	0.16 (0.26)	28 (17.1)	239 (24.0)	398 (26.6)
8+	0.21 (0.27)	42 (25.6)	427 (42.8)	407 (27.2)
Medications prescribed at discharge				
0‐4	0.22 (0.34)	56 (34.2)	120 (12.0)	337 (22.6)
5‐6	0.18 (0.30)	38 (23.2)	170 (17.1)	360 (24.1)
7‐9	0.18 (0.29)	40 (24.4)	274 (27.5)	461 (30.7)
10+	0.24 (0.28)	30 (18.3)	433 (43.4)	336 (22.5)
Total number of changes				
1	0.27 (0.44)	63 (38.4)	0 (‐)	172 (11.5)
2‐3	0.21 (0.31)	70 (42.7)	266 (26.7)	565 (37.8)
4‐5	0.18 (0.25)	16 (9.8)	323 (32.4)	414 (27.7)
6+	0.21 (0.28)	15 (9.2)	408 (40.9)	343 (23.0)
New medications				
0	0.24 (0.38)	32 (19.5)	33 (3.3)	119 (7.8)
1	0.23 (0.34)	53 (32.3)	131 (13.1)	290 (19.4)
2‐3	0.18 (0.27)	52 (71.7)	443 (44.3)	669 (44.8)
4+	0.21 (0.27)	27 (16.5)	390 (39.1)	416 (27.8)
Discontinued medications				
0	0.25 (0.34)	141 (86.0)	477 (47.8)	828 (55.4)
1+	0.15 (0.23)	23 (14.0)	520 (52.2)	666 (44.6)
Modified medications				
0	0.21 (0.33)	129 (78.7)	507(50.9)	1012 (67.7)
1+	0.20 (0.25)	35 (21.3)	490 (49.2)	482 (32.3)
Continued medications				
0	0.26 (0.37)	64 (39.0)	216 (21.7)	381 (25.5)
1‐3	0.16 (0.26)	27 (16.5)	205 (20.6)	379 (25.4)
4‐6	0.17 (0.26)	30 (18.3)	261 (26.2)	420 (28.1)
7+	0.23 (0.29)	43 (26.2)	315 (31.6)	214 (21.0)

Overall, 860 (32.4%) patients were readmitted to hospital, visited the emergency department, or died in the 30 days postdischarge; 696 events (80% of all events) were ED visits, 153 (18%) were re‐hospitalizations, and 14 (1.6%) were deaths. The incidence rate for the composite outcome, over a total of 65 560 days of follow‐up, was 1.31 events/100 person‐days. Some of the most common reasons for readmission or ED visit included abdominal pain, dyspnea and respiratory abnormalities, cellulitis, and heart failure.

**Figure 2:**
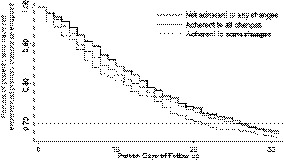
Kaplan‐Meier curves for probability of not experiencing the primary composite endpoint 30 days postdischarge according to level of adherence

Kaplan‐Meier survival curves indicated that the probability of remaining event free 30 days postdischarge was lowest for those who were not adherent to any changes and highest among patients who were adherent to all medication changes across the follow‐up period (Figure 2). This finding was confirmed by the results of the multivariable time‐varying Cox models. After adjustment for confounders, patients who were not adherent to any of their medication changes had a 35% higher risk of adverse events compared to those adherent to all medication changes (1.41 vs 1.27 events/100 person‐days, adjusted hazard ratio [aHR]: 1.35, 95% CI: 1.06‐1.71). Additionally, those who were adherent to some of their medication changes at discharge had a 10% elevated risk of adverse events in 30 days postdischarge compared to those who were adherent to all medication changes (1.31 vs 1.27 events/100 person‐days); however, the confidence interval did include the null ([aHR]: 1.10, 95% CI: 0.94‐1.30) (Table 3). There was also a trend towards an increased risk of adverse events with each 10% increase in the percent of nonadherence, modeled as a continuous time‐dependent variable (aHR: 1.02, 95% CI: 0.99‐1.04).

Secondary analyses suggested that the impact of nonadherence on adverse events was mainly driven by new medications which were not filled. Whereas not filling any new medications was associated with an increased risk of adverse events (aHR:1.34, 95% CI: 1.08‐1.66), no increased risk was observed for nonadherence to discontinuations (aHR: 0.86, 95% CI: 0.54‐1.35) or dose changes (aHR: 0.89, 95% CI: 0.64‐1.21). The magnitude and direction of the association between nonadherence and adverse events did not change substantially in different sensitivity analyses (a) after the follow‐up period was extended to 90 days, (b) when early events were excluded, (c) when intervention and control patients were analyzed separately, (d) when total out‐of‐pocket medication costs were included as a confounder in the model, (e) when we adjusted for a time‐varying indicator of postdischarge visits with a community‐based physician, or (f) when we adjusted for the time‐varying cumulative number of visits (Appendix S1: Table S2). Additionally, the proportion of medication changes which were not adhered to per patient per day among those who ultimately had a visit with a community physician was very similar in the time period prior to and following the visit (Appendix S1: Figures S1 and S2).

## 4 DISCUSSION

In our prospective cohort study of hospitalized medical and surgical patients, we found that almost half of patients were nonadherent to at least one medication change made at discharge in the 30 days posthospitalization and that patients who were not adherent to any of their medication changes had a significantly higher risk of adverse events compared to those who were adherent to all changes. Although the crude incidence of adverse events in 30 days for those who were nonadherent to some medication changes was higher than for those adherent to all changes, and the hazard ratio suggested an increased in risk for adjusted models, the confidence interval for the estimate included the null. Moreover, we found that the adverse effects of nonadherence were mainly driven by not filling newly prescribed medications.

**Table 3:** Association between nonadherence to medication changes and adverse events in 30‐days post discharge

Exposure	Events	Person‐days	Incidence rate^a^ (95% CI)	Adjusted^b^ HR (95% CI)
Adherent to all changes	226	17 802	1.27 (1.11‐1.44)	Reference
Adherent to some of the changes	520	39 702	1.31 (1.20‐1.43)	1.10 (0.94‐1.30)
Not adherent to any of the changes	114	8 086	1.41 (1.12‐1.69)	1.35 (1.06‐1.71)

^a^ Events/100 person‐days.

^b^ Adjusted for number of preadmission prescribers, whether the patient had at least one ED visit prior to admission, the unit the patient was discharged from, presence of cardiac arrhythmia, or metastatic cancer and the time‐varying number of new medications filled with high risk of harm.

To our knowledge, only two other studies have evaluated the impact of nonadherence to medications prescribed at hospital discharge. A study by Coleman et al found that 14% of older adults discharged from hospital who were nonadherent to prescribed medications were re‐hospitalized in 30 days compared to 6% who were adherent;^42^ however, only unadjusted risks were reported. Moreover, a study analyzing patients with acute myocardial infarction discharged from hospital found that only 25% of patients filled all new medications prescribed at hospital discharge and that the adjusted 1‐year mortality rate was higher in patients who did not fill any of their discharge medications compared to those who filled all. This study was similar to our own in that it compared hospital discharge prescriptions to medication dispensing data; however, it was restricted to patients with AMI.^15^

Importantly, we found that patients who did not fill any of their newly prescribed medications were at the highest risk of adverse events after hospitalization. It is possible that nonadherence may be a marker for worse outcomes as a result of specific health behaviors.^43^ Indeed, starting a new medication involves a patient's personal values and expectations around the perceived safety and effectiveness of medication treatment overall as well as their own confidence and self‐efficacy.^44^ Combined, the consistency of findings with our current study supports the development of policy and health system interventions to reduce nonadherence among patients who are discharged from hospital. Indeed, a recent randomized controlled trial of a health system intervention that involved medication review, motivational interviews with patients, and follow‐up with the primary care physician, pharmacy, and nursing home found that in medical patients using 5+ medications, the risk of readmission was reduced by 38% compared to those receiving usual care.^45^ This intervention, however, was very resource intensive and required a significant amount of clinician oversight. Most organizations thinking of implementing a similar type of intervention would have difficulty offering it to all hospitalized patients on a larger scale.

In an effort to address the challenge of medication nonadherence following discharge through a pragmatic, scalable intervention, our team developed and is in the process of piloting a mobile application to enhance medication management following hospital discharge called Smart About Meds (SAM). The application retrieves medications prescribed to a patient from the discharge prescription as well as dispensed medications via real‐time linkage with the provincial pharmacy claims database. The app creates a patient‐friendly list of prescribed/dispensed medications, grouped by therapeutic class, and offers tools targeting barriers to adherence. These tools include but are not limited to: (a) integrated adherence monitoring and feedback (alerts patients, caregivers, and hospital pharmacists to adherence problems), (b) pharmacy connect (allows patients to connect with hospital pharmacist through secure messaging service), and (c) symptom checker (allows patients to determine which of their medications has side effects similar to experienced symptoms).

Although SAM is less resource intensive than the multifaceted intervention developed by Ravn‐Nielsen et al, most organizations would likely still have difficulty offering it to all patients being discharged from hospital. Therefore, the ability to identify those at highest risk of nonadherence who may benefit the most from receiving such an intervention is essential. The results of a previous study by our research team on the patient‐ and medication‐level factors associated with nonadherence in the postdischarge period provide important insights into the characteristics of these high‐risk patients. Our study suggested that failure to follow medication changes was highest for dose increases, symptom relief medications, those that require prior authorization, and medications that had not been administered during the hospital stay. At the patient level, those with at least one preadmission hospitalization, who did not have any medications dispensed prior to admission, and were discharged from thoracic surgery or to a long‐term care facility, also had a higher risk of failure to follow changes.^46^ Overall, we hypothesize that a pragmatic medication‐related intervention (such as SAM) targeted to high‐risk patients has the potential to improve health outcomes for these patients in the postdischarge period and is feasible for health care organizations with limited resources to implement.

### 4.1 Limitations

There are limitations to keep in mind when interpreting the results of this study. First, we used dispensing data to measure adherence in the postdischarge period and could not actually observe the medications patients were taking. This could mean that although patients are filling their medications, they may not be taking them, or taking them at the wrong dose. Moreover, even if a patient does not fill discontinued medications, they may still be taking a supply that was left over prior to admission.^22^, ^47^, ^48^ Therefore, this could be a source of potential unmeasured confounding. Additionally, as is the case in any study which evaluates the association between medication adherence and health outcomes, diet, lifestyle, and social determinants of health are important factors we did not have access to information on which could also be a source of unmeasured confounding.

For some medications, accurately calculating daily dose from dispensing data can be challenging, potentially overestimating nonadherence to dose changes. Last, we did not measure the appropriateness of the changes made to medications in‐hospital. It is quite possible that changes were not entirely appropriate for that patient at that time and may have been reversed by the patient's community‐based physician after discharge from hospital. However, we did adjust for the risk of harm associated with medications that were filled in an attempt to address this issue and also found that the incidence of nonadherence did not differ between the time period prior to a visit with the community physician or after.

## 5 CONCLUSIONS

In summary, we found that almost half of patients were not adherent to some or all changes made to their medications at hospital discharge and that nonadherence to all changes was associated with an increased risk of adverse events. Health policy and patient interventions aimed at barriers to adherence have the potential to reduce adverse health outcome for patients as well as the burden on the health care system in terms of both cost and utilization.

## Supporting information

Supplementary Material

Appendix S1

## References

[1] Higashi T , Wenger NS , Adams JL , et al. Relationship between number of medical conditions and quality of care. N Engl J Med. 2007;356:2496‐2504. 17568030 10.1056/NEJMsa066253

[2] Weir DL , Majumdar SR , McAlister FA , Marrie TJ , Eurich DT . The impact of multimorbidity on short‐term events in patients with community‐acquired pneumonia: prospective cohort study. Clin Microbiol Infect. 2015;21:264.e7‐264.e13. 10.1016/j.cmi.2014.11.002 25658532

[3] Basu J , Avila R , Ricciardi R . Hospital readmission rates in U.S. States: are readmissions higher where more patients with multiple chronic conditions cluster? Health Serv Res. 2016;51:1135‐1151. 26481190 10.1111/1475-6773.12401 PMC4874830

[4] Dattalo M , DuGoff E , Ronk K , Kennelty K , Gilmore‐Bykovskyi A , Kind AJ . Apples and oranges: four definitions of multiple chronic conditions and their relationship to 30‐day hospital readmission. J Am Geriatr Soc. 2017;65:712‐720. 28205206 10.1111/jgs.14539 PMC5397355

[5] Hijazi HH , Alyahya MS , Hammouri HM , Alshraideh HA . Risk assessment of comorbidities on 30‐day avoidable hospital readmissions among internal medicine patients. J Eval Clin Pract. 2017;23:391‐401. 27576302 10.1111/jep.12631

[6] Smith SM , Soubhi H , Fortin M , Hudon C , O’Dowd T . Managing patients with multimorbidity: systematic review of interventions in primary care and community settings. BMJ. 2012;345:e5205. 22945950 10.1136/bmj.e5205 PMC3432635

[7] Marengoni A , Pasina L , Concoreggi C , et al. Understanding adverse drug reactions in older adults through drug–drug interactions. Eur J Intern Med. 2014;25:843‐846. 25312593 10.1016/j.ejim.2014.10.001

[8] Slight SP , Seger DL , Franz C , Wong A , Bates DW . The national cost of adverse drug events resulting from inappropriate medication‐related alert overrides in the United States. J Am Med Inform Assoc. 2018;25:1183‐1188. 29939271 10.1093/jamia/ocy066 PMC7646874

[9] Wu C , Bell CM , Wodchis WP . Incidence and economic burden of adverse drug reactions among elderly patients in Ontario Emergency Departments. Drug Saf. 2012;35:769‐781. 22823502 10.1007/BF03261973 PMC3714138

[10] Forster AJ , Murff HJ , Peterson JF , Gandhi TK , Bates DW . Adverse drug events occurring following hospital discharge. J Gen Intern Med. 2005;20:317‐323. 15857487 10.1111/j.1525-1497.2005.30390.x PMC1490089

[11] Harris CM , Sridharan A , Landis R , Howell E , Wright S . What happens to the medication regimens of older adults during and after an acute. Hospitalization? J Patient Saf. 2013;9:150‐153. 23965837 10.1097/PTS.0b013e318286f87d

[12] Viktil KK , Blix HS , Eek AK , Davies MN , Moger TA , Reikvam A . How are drug regimen changes during hospitalisation handled after discharge: a cohort study. BMJ Open. 2012;2:e001461. 10.1136/bmjopen-2012-001461 PMC3532967 23166124

[13] Cochrane RA , Mandal AR , Ledger‐Scott M , Walker R . Changes in drug treatment after discharge from hospital in geriatric patients. BMJ. 1992;305:694‐696. 1393119 10.1136/bmj.305.6855.694 PMC1882955

[14] Fallis BA , Dhalla IA , Klemensberg J , Bell CM . Primary medication non‐adherence after discharge from a general internal medicine service. PLoS ONE. 2013;8:e61735. 23658698 10.1371/journal.pone.0061735 PMC3642181

[15] Jackevicius CA , Li P , Tu JV . Prevalence, predictors, and outcomes of primary nonadherence after acute myocardial infarction. Circulation. 2008;117:1028‐1036. 18299512 10.1161/CIRCULATIONAHA.107.706820

[16] Mulhem E , Lick D , Varughese J , Barton E , Ripley T , Haveman J . Adherence to medications after hospital discharge in the elderly. Int J Fam Med. 2013;2013:1‐6. 10.1155/2013/901845 PMC3622370 23589775

[17] Himmel W , Tabache M , Kochen MM . What happens to long‐term medication when general practice patients are referred to hospital? Eur J Clin Pharmacol. 1996;50:253‐257. 8803514 10.1007/s002280050103

[18] Tamblyn R , Huang AR , Meguerditchian AN , et al. Using novel Canadian resources to improve medication reconciliation at discharge: study protocol for a randomized controlled trial. Trials. 2012;13:150. 22920446 10.1186/1745-6215-13-150 PMC3502593

[19] Tamblyn R , Winslade N , Lee TC , et al. Improving patient safety and efficiency of medication reconciliation through the development and adoption of a computer‐assisted tool with automated electronic integration of population‐based community drug data: the RightRx project. J Am Med Inform Assoc. 2018;25:482‐495. 29040609 10.1093/jamia/ocx107 PMC6018649

[20] Tamblyn R , Lavoie G , Petrella L , Monette J . The use of prescription claims databases in pharmacoepidemiological research: the accuracy and comprehensiveness of the prescription claims database in Quebec. J Clin Epidemiol. 1995;48:999‐1009. 7775999 10.1016/0895-4356(94)00234-h

[21] Wilchesky M , Tamblyn RM , Huang A . Validation of diagnostic codes within medical services claims. J Clin Epidemiol. 2004;57:131‐141. 15125622 10.1016/S0895-4356(03)00246-4

[22] Tamblyn R , Eguale T , Huang A , Winslade N , Doran P . The incidence and determinants of primary nonadherence with prescribed medication in primary care: a cohort study. Ann Intern Med. 2014;160:441‐450. 24687067 10.7326/M13-1705

[23] Tamblyn R , Poissant L , Huang A , et al. Estimating the information gap between emergency department records of community medication compared to on‐line access to the community‐based pharmacy records. J Am Med Inform Assoc JAMIA. 2014;21:391‐398. 23956015 10.1136/amiajnl-2013-001704 PMC3994851

[24] Sketris IS , Metge C , Shevchuk Y , et al. Comparison of anti‐infective drug use in elderly persons in Manitoba, Nova Scotia, and Saskatchewan, Canada: Relationship to drug insurance reimbursement policies. Am J Geriatr Pharmacother. 2004;2:24‐35. 15555476 10.1016/s1543-5946(04)90004-9

[25] Bernstein C . The association between corticosteroid use and development of fractures among IBD patients in a population‐based database. Am J Gastroenterol. 2003;98:1797‐1801. 12907335 10.1111/j.1572-0241.2003.07590.x

[26] Metge C , Black C , Peterson S , Kozyrskyj AL . The population’s use of pharmaceuticals. Med Care. 1999;37:JS42‐59. 10409017 10.1097/00005650-199906001-00008

[27] Wertheimer AI . The defined daily dose system (DDD) for drug utilization review. Hosp Pharm. 1986;21:233‐234, 239–241, 258. 10317694

[28] Gupta S , McColl MA , Guilcher SJ , Smith K . Cost‐related nonadherence to prescription medications in Canada: a scoping review. Patient Prefer Adherence. 2018;12:1699‐1715. 30233150 10.2147/PPA.S170417 PMC6134942

[29] Morgan SG , Lee A . Cost‐related non‐adherence to prescribed medicines among older adults: a cross‐sectional analysis of a survey in 11 developed countries. BMJ Open. 2017;7:e014287. 10.1136/bmjopen-2016-014287 PMC5293866 28143838

[30] Pasina L , Brucato AL , Falcone C , et al. Medication Non‐adherence among elderly patients newly discharged and receiving polypharmacy. Drugs Aging. 2014;31:283‐289. 24604085 10.1007/s40266-014-0163-7

[31] Stewart S , Pearson S . Uncovering a multitude of sins: medication management in the home post acute hospitalisation among the chronically ill. Aust N Z J Med. 1999;29:220‐227. 10342021 10.1111/j.1445-5994.1999.tb00687.x

[32] Mansur N , Weiss A , Beloosesky Y . Is there an association between inappropriate prescription drug use and adherence in discharged elderly patients? Ann Pharmacother. 2009;43:177‐184. 19193583 10.1345/aph.1L461

[33] Mansur N , Weiss A , Beloosesky Y . Relationship of in‐hospital medication modifications of elderly patients to postdischarge medications, adherence, and mortality. Ann Pharmacother. 2008;42:783‐789. 18445704 10.1345/aph.1L070

[34] Canadian Institute for Health Information . Indicator Library: General Methodology Notes — Clinical Indicators. Ottawa, ON: CIHI; 2018.

[35] Gray SL , Mahoney JE , Blough DK . Medication adherence in elderly patients receiving home health services following hospital discharge. Ann Pharmacother. 2001;35:539‐545. 11346058 10.1345/aph.10295

[36] Quan H , Sundararajan V , Halfon P , et al. Coding algorithms for defining comorbidities in ICD‐9‐CM and ICD‐10 administrative data. Med Care. 2005;43:1130‐1139. 16224307 10.1097/01.mlr.0000182534.19832.83

[37] Nikesh P , Khalid A , Stevenson JM , et al. Incidence and cost of medication harm in older adults following hospital discharge: a multicentre prospective study in the UK. Br J Clin Pharmacol. 2018 https://bpspubs.onlinelibrary.wiley.com/doi/abs/10.1111/bcp.13613 10.1111/bcp.13613 PMC6046489 29790202

[38] Akaike H . A new look at the statistical model identification. Autom Control IEEE Trans On. 1974;19:716‐723.

[39] Greenland S , Daniel R , Pearce N . Outcome modelling strategies in epidemiology: traditional methods and basic alternatives. Int J Epidemiol. 2016;45:565‐575. 27097747 10.1093/ije/dyw040 PMC4864881

[40] Abrahamowicz M , du Berger R , Grover SA . Flexible modeling of the effects of serum cholesterol on coronary heart disease mortality. Am J Epidemiol. 1997;145:714‐729. 9125998 10.1093/aje/145.8.714

[41] Abrahamowicz M , MacKenzie TA . Joint estimation of time‐dependent and non‐linear effects of continuous covariates on survival. Stat Med. 2007;26:392‐408. 16479552 10.1002/sim.2519

[42] Coleman EA , Smith JD , Raha D , Min S . Posthospital medication discrepancies: prevalence and contributing factors. Arch Intern Med. 2005;165:1842‐1847. 16157827 10.1001/archinte.165.16.1842

[43] Simpson SH . A meta‐analysis of the association between adherence to drug therapy and mortality. BMJ. 2006;333:15. 16790458 10.1136/bmj.38875.675486.55 PMC1488752

[44] Lehane E , McCarthy G . Intentional and unintentional medication non‐adherence: A comprehensive framework for clinical research and practice? A discussion paper. Int J Nurs Stud. 2007;44:1468‐1477. 16973166 10.1016/j.ijnurstu.2006.07.010

[45] Ravn‐Nielsen LV , Duckert M‐L , Lund ML , et al. Effect of an In‐hospital multifaceted clinical pharmacist intervention on the risk of readmission: a randomized clinical trial. JAMA Intern Med. 2018;178:375‐382. 29379953 10.1001/jamainternmed.2017.8274 PMC5885912

[46] Weir DL , Motulsky A , Abrahamowicz M , et al. Challenges at care transitions: failure to follow medication changes made at hospital discharge. Am J Med. 2019;132:1216‐1224.e5. 31145881 10.1016/j.amjmed.2019.05.003

[47] Gardner TL , Dovey SM , Tilyard MW , Gurr E . Differences between prescribed and dispensed medications. N Z Med J. 1996;109:69‐72. 8606821

[48] Nilsson JLG , Johansson H , Wennberg M . Large differences between prescribed and dispensed medicines could indicate undertreatment. Drug Inf J. 1995;29:1243‐1246.

