# Supplementary material for: Failure to follow medication changes made at hospital discharge is associated with adverse events in 30 days
Source: Health Serv Res. 2020 May 20;55(4):512–23. doi: 10.1111/1475-6773.13292 (PMC7376001; doi:10.1111/1475-6773.13292)
Supplement: Supplementary file 2 — Appendix S1 [file HESR-55-512-s002.docx]

### Appendix S1

**Table S1**. List of excluded medications

| **ingredient** |
| --- |
| ACAMPROSATE |
| ALGINIC-ACID |
| ALUMINUM |
| AMCINONIDE |
| AMPHOTERICIN-B |
| ANAKINRA |
| ANETHOLTRITHIONE |
| ARTIFICIAL-SALIVA |
| ARTIFICIAL-TEARS-MISCELLANEOUS |
| ASA |
| ATROPINE |
| AZELAIC-ACID |
| BACITRACIN |
| BELIMUMAB |
| BENZOCAINE |
| BENZYDAMINE |
| BETAMETHASONE-DIPROPIONATE |
| BETAMETHASONE-DIPROPIONATE/PROPYLENE-GLYCOL |
| BETAMETHASONE-VALERATE |
| BETAXOLOL |
| BIFIDOBACTERIUM |
| BIMATOPROST |
| BISACODYL |
| BISMUTH-SUBSALICYLATE |
| BRIMONIDINE |
| BRINZOLAMIDE |
| BROMOCRIPTINE |
| BUPRENORPHINE |
| BUTALBITAL |
| CADEXOMER-IODINE |
| CAFFEINE |
| CALAMINE |
| CALCIPOTRIOL |
| CALCIUM-CARBONATE |
| CALCIUM-CARBONATE-ANTACID |
| CALCIUM-CITRATE |
| CALCIUM-SALTS |
| CAMPHOR |
| CAPSAICIN |
| CARBOMER |
| CARBOXYMETHYLCELLULOSE |
| CARBOXYMETHYLCELLULOSE-DRESSINGS |
| CEFTAZIDIME |
| CETIRIZINE |
| CETUXIMAB |
| CHLORAL-HYDRATE |
| CHLORHEXIDINE |
| CICLOPIROX |
| CILASTATIN |
| CLOBETASOL-PROPIONATE |
| CLORAZEPATE |
| CLOTRIMAZOLE |
| COLLAGENASE |
| CROMOGLYCATE-SODIUM |
| CYCLOMETHICONE |
| CYCLOPENTOLATE |
| CYPROHEPTADINE |
| CYPROTERONE |
| DAPTOMYCIN |
| DEFERASIROX |
| DESLORATADINE |
| DESONIDE |
| DESOXIMETASONE |
| DESVENLAFAXINE |
| DEXTRAN |
| DEXTROMETHORPHAN |
| DEXTROSE |
| DIBASIC-SODIUM-PHOSPHATE |
| DIBUCAINE |
| DICHLOROBENZENE |
| DICYCLOMINE |
| DIFLUCORTOLONE-VALERATE |
| DIFLUPREDNATE |
| DIMETHICONE |
| DIPHENHYDRAMINE |
| DOCETAXEL |
| DOCUSATE-CALCIUM |
| DOCUSATE-SODIUM |
| DORZOLAMIDE |
| EFINACONAZOLE |
| EMPAGLIFLOZIN |
| ENTERAL-NUTRITION |
| ERYTHROMYCIN-BASE |
| ERYTHROMYCIN-GLUCEPTATE |
| ERYTHROMYCIN-STEARATE |
| ESCITALOPRAM |
| ESTROGENS |
| ESTROGENS-CONJUGATED-BIOLOGIC |
| ESTROGENS-CONJUGATED-SYNTHETIC |
| ETONOGESTREL |
| EUCALYPTOL-OIL |
| EUCERIN |
| EXENATIDE |
| FAMOTIDINE |
| FERROUS-FUMARATE |
| FERROUS-GLUCONATE |
| FERROUS-SULFATE |
| FLUOCINOLONE-ACETONIDE |
| FLUOCINONIDE |
| FLUORINE |
| FLUOROMETHOLONE |
| FLUOROURACIL |
| FLUTICASONE-FUROATE |
| FLUTICASONE-FUROATE-NASAL |
| FOLIC-ACID |
| FOSFOMYCIN |
| FRAMYCETIN |
| FULVESTRANT |
| FUSIDIC-ACID |
| GATIFLOXACIN |
| GEL-LUBRICANT |
| GLUCOSAMINE |
| GLUCOSE-POLYMERS |
| GLYCERIN |
| GRAMICIDIN |
| HALOBETASOL-PROPIONATE |
| HEPATITIS-A-VACCINE |
| HEPATITIS-B-VACCINE |
| HOMATROPINE |
| HYDROCORTISONE-ACETATE |
| HYDROCORTISONE-VALERATE |
| HYDROGEL |
| HYDROXYPROPYLMETHYLCELLULOSE |
| IBRUTINIB |
| IBUPROFEN |
| IMIPENEM |
| IRON-POLYPEPTIDE |
| IRON-SALTS |
| IRON-SUCROSE |
| ISOSORBIDE-DINITRATE |
| IVERMECTIN |
| KETOROLAC |
| KETOTIFEN |
| LACOSAMIDE |
| LACTASE |
| LACTOBACILLUS |
| LACTULOSE |
| LANOLIN |
| LATANOPROST |
| LAXATIVES-NATURALS-MISCELLANEOUS |
| LEVOBUNOLOL |
| LINACLOTIDE |
| LOPERAMIDE |
| LORATADINE |
| LOTEPREDNOL |
| LURASIDONE |
| LUTEIN |
| MACITENTAN |
| MAGNESIA |
| MAGNESIUM |
| MAGNESIUM-SALTS |
| MAPROTILINE |
| MEGESTROL |
| MELATONIN |
| MENTHOL |
| METHYLPREDNISOLONE |
| METHYL-SALICYLATE |
| MICONAZOLE |
| MINERAL-OIL |
| MINERAL-OIL (HEAVY) |
| MINERALS-MULTIVALENT |
| MOISTURIZER-MISCELLANEOUS |
| MOMETASONE-FUROATE |
| MOMETASONE-NASAL |
| MOUTHWASH-MISCELLANEOUS |
| MULTIVITAMINS |
| MUPIROCIN |
| NALOXONE |
| NALTREXONE |
| NAPROXEN-SODIUM |
| NEOMYCIN |
| NEPAFENAC |
| NIACIN |
| NICOTINE |
| NORGESTIMATE |
| OATS-COLLOIDAL |
| OFLOXACIN |
| OLOPATADINE |
| OMALIZUMAB |
| OMEGA-3 OILS |
| ORPHENADRINE-CITRATE |
| PANCRELIPASE |
| PARAFFIN |
| PARAFFIN-SOFT |
| PEG-ELECTROLYTES |
| PENICILLIN-G-BENZATHINE |
| PENTAZOCINE |
| PERMETHRIN |
| PERPHENAZINE |
| PETROLATUM-WHITE |
| PHENELZINE |
| PILOCARPINE-HYDROCHLORIDE |
| PINAVERIUM |
| PIRFENIDONE |
| PNEUMOCOCCUS-VACCINE |
| POLYETHYLENE-GLYCOL |
| POLYETHYLENE-GLYCOL 3350 |
| POLYMYXIN-B |
| POLYSORBATE-80 |
| POLYVINYL-ALCOHOL |
| POTASSIUM-CHLORIDE |
| POTASSIUM-CITRATE |
| POVIDONE |
| POVIDONE-IODINE |
| PRAMOXINE |
| PREDNISOLONE |
| PRILOCAINE |
| PRIMAQUINE |
| PROBIOTIC |
| PSYLLIUM |
| PYRITHIONE-ZINC |
| RESORCINOL |
| ROFLUMILAST |
| SALICYLATE-DIETHYLAMINE |
| SALICYLIC-ACID |
| SALT-WATER-DESALINATED |
| SELEGILINE |
| SELENIUM-SULFIDE |
| SENNOSIDES-A-B |
| SHORT-RAGWEED-ORAL |
| SILVER-NITRATE |
| SILVER-SULFADIAZINE |
| SIMETHICONE |
| SIMPLE-TOPICAL-BASE |
| SOAPS-MISCELLANEOUS |
| SODIUM-BICARBONATE |
| SODIUM-CHLORIDE |
| SODIUM-PHOSPHATE/BIPHOSPHATE |
| SULFACETAMIDE |
| SULFUR |
| SUNSCREENS-MISCELLANEOUS |
| TAPENTADOL |
| TAR-MINERAL |
| THYROID |
| TOCOPHERYL |
| TRAVOPROST |
| TRETINOIN |
| TRIAMCINOLONE-ACETONIDE-TOPICAL |
| TRIAMCINOLONE-BUCAL |
| TRIAMCINOLONE-NASAL |
| TULLES-PARAFIN |
| UBIDECARENONE |
| ULIPRISTAL |
| UREA |
| VARDENAFIL |
| VARICELLA-ZOSTER-VACCINE |
| VEDOLIZUMAB |
| VITAMIN-A |
| VITAMIN-B1 |
| VITAMIN-B12 |
| VITAMIN-B2 |
| VITAMIN-C |
| VITAMIN-D |
| VITAMIN-D2 |
| VITAMIN-E |
| VORTIOXETINE |
| ZINC-GLUCONATE |
| ZINC-OXIDE |
| ZINC-SULFATE |
| ZOLPIDEM |
| ZOPICLONE |

**Schematic 1.** Daily dose calculations for prescribed and dispensed medications

The daily dose for prescribed medications was based on the dose and frequency information abstracted from the discharge prescription from the patients chart.

If a patient was prescribed clopidegrel 75 mg PO DAILY, the daily dose would be calculated as the following:

Daily dose prescribed= 75mg* (1 time/day)=75 mg/day

The daily dose for dispensed medication was based on the quantity, days supply, strength, ingredient and dispensing date fields from pharmacy claims data. Each medication dispensation for each patient was identified based on their unique healthcare beneficiary number.

If a patient was dispensed clopidegrel on the day after they were discharged from hospital, where the strength was 75mg tablets, the quantity was 30 tablets and the duration or days supply was 30 days, the daily dose would be calculated as the following:

Daily dose dispensed= $\frac{\left( 75\frac{mg}{tablet} \right)*(30 tablets)}{30 dats}$=75 mg/day

|  | **Exposure** | **Adjusted hazard ratio** | **95% confidence interval** |
| --- | --- | --- | --- |
| ***Main analysis***  ***(n=2,655 patients)*** |  |  |  |
|  | Adherent all | ***Reference*** |  |
|  | Adhere Some | 1.11 | 0.94-1.30 |
|  | Adhere none | 1.35 | 1.06-1.71 |
| ***Start follow-up day 3, 30 days follow-up total***  ***(n=2,521)*** |  |  |  |
|  | Adherent all | ***Reference*** |  |
|  | Adhere Some | 1.03 | 0.87-1.23 |
|  | Adhere none | 1.17 | 0.90-1.54 |
| ***Extend follow-up time to 90-days***  ***(n=2,655)*** |  |  |  |
|  | Adherent all | ***Reference*** |  |
|  | Adhere Some | 1.08 | 0.95-1.23 |
|  | Adhere none | 1.35 | 1.12-1.63 |
| ***Start follow-up on day 3 & extend follow up to 90-days (n=2,521)*** |  |  |  |
|  | Adherent all | ***Reference*** |  |
|  | Adhere Some | 1.04 | 0.91-1.52 |
|  | Adhere none | 1.24 | 1.01-1.52 |
| ***Adjusted for time-varying indicator of physician visits (n=2,655 patients)*** |  |  |  |
|  | Adherent all | ***Reference*** |  |
|  | Adhere Some | 1.11 | 0.95-1.62 |
|  | Adhere none | 1.27 | 1.00-1.62 |
| ***Adjusted for time-varying cumulative number of visits (n=2,655 patients)*** |  |  |  |
|  | Adherent all | ***Reference*** |  |
|  | Adhere Some | 1.12 | 0.96-1.64 |
|  | Adhere none | 1.25 | 0.99-1.60 |
| ***Intervention patients (n=1,178)*** |  |  |  |
|  | Adherent all | ***Reference*** |  |
|  | Adhere Some | 1.25 | 0.97-1.64 |
|  | Adhere none | 1.42 | 0.96-2.11 |
| ***Control patients (n=1,477)*** |  |  |  |
|  | Adherent all | ***Reference*** |  |
|  | Adhere Some | 1.00 | 0.81-1.23 |
|  | Adhere none | 1.20 | 0.90-1.60 |
| ***Adjusted for out of pocket medication costs***  ***(n=2,655 patients)*** |  |  |  |
|  | Adherent all | ***Reference*** |  |
|  | Adhere Some | 1.11 | 0.94-1.30 |
|  | Adhere none | 1.35 | 1.06-1.71 |

**Table S2:** Results of sensitivity analyses

**Figure S1.** Proportion of medication changes not adhered to per day per patient in those who have at least one post discharge physician visit, restricted to person time ***prior to*** visit with lowess non-parametric smoother

**
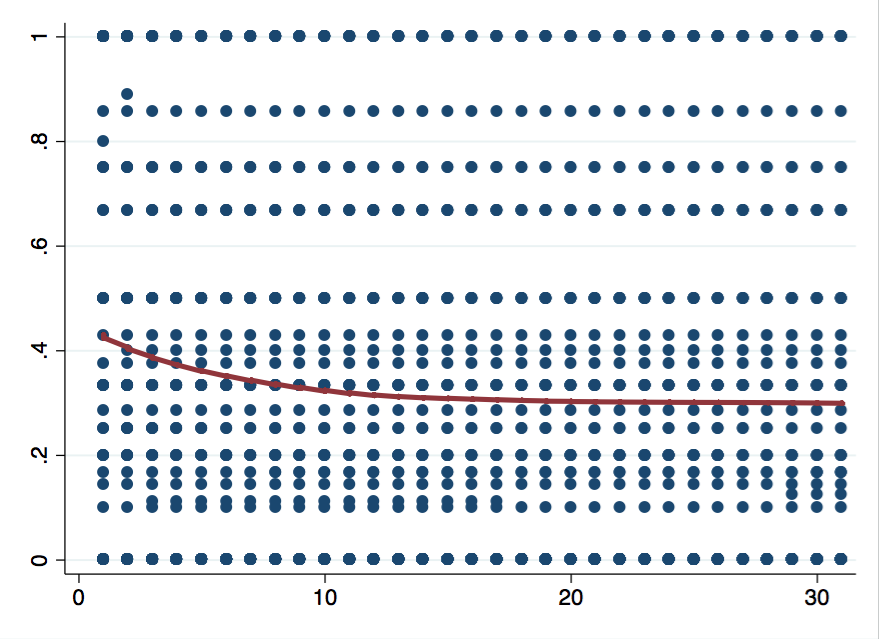
**

Proportion of medication changes not adhered to per patient

Days of follow-up

**Figure S2.** Proportion of medication changes not adhered to per day per patient in those who have at least one post discharge physician visit, restricted to person time ***after*** visit with lowess non-parametric smoother

**
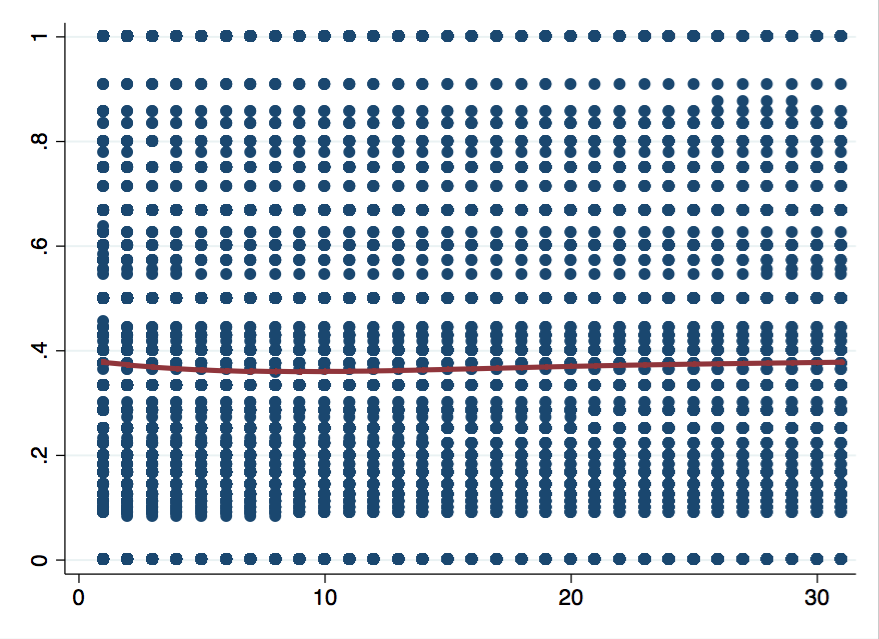
**

Proportion of medication changes not adhered to per patient

Days of follow-up
